# The Pleiotropic Role of the MicroRNA-17-92 Cluster in Cardiovascular Diseases and Cancer

**DOI:** 10.31083/RCM27966

**Published:** 2025-05-27

**Authors:** Arun Samidurai, Manu Saravanan, Virginia Villani Zwiren, Varun Kodali, Kush Savsani, Nuhash Bhuiyan, Shruti Yerramothu, Suet Ying Valerie Lau, Ena Baral, Sara Hammel, Anindita Das

**Affiliations:** ^1^Division of Cardiology, Pauley Heart Center, Internal Medicine, Virginia Commonwealth University, Richmond, VA 23298, USA

**Keywords:** miRNAs, miRNA-17-92, cardiovascular diseases, cancer, biomarker, therapeutic targets

## Abstract

Cardiovascular diseases, including acute myocardial infarctions, heart failure, hypertension, adverse cardiac remodeling, hypertrophy, atherosclerosis, and coronary artery disease, continue to lead to global mortality rates. Annual global cancer mortality rates follow closely behind, emphasizing the need to develop novel therapeutic approaches. MicroRNAs (miRNAs), a class of short non-coding RNAs, regulate cascades of signaling pathways and their downstream targets, exerting control over numerous biological processes. Dysregulation in specific miRNAs is linked to various pathogenesis, including cancer and cardiovascular disease. Among these miRNAs, the miRNA-17-92 cluster plays versatile roles at the nexus of critical physiological and pathological processes, including cardiac diseases and malignancy. This review aimed to provide a holistic analysis of the current progress in identifying, developing, and utilizing the miRNA-17-92 cluster to combat cardiovascular diseases and cancer. The members of the miRNA-17-92 cluster exert control over numerous cellular pathways that regulate, suppress, and promote various aspects of cardiomyocyte differentiation, regeneration, and aging. Certain pathways controlled by the cluster are protective when properly expressed. Others can propagate unchecked cardiovascular disease progression and mortality due to poorly controlled over/under-regulation. Similarly, the miRNA-17-92 cluster plays critical regulatory roles in the occurrence, metastasis, and prognosis of multiple cancers, which may allow the cluster to serve as diagnostic and prognostic biomarkers of malignancy. This review provides a brief overview of the multifaceted roles of the miRNA-17-92 cluster to deliver some insight into the development of novel targeted therapeutics for cardiovascular diseases and cancer via controlling the expression of specific subsets within this cluster. Additionally, this review systematically summarizes the established molecular mechanisms of the miRNA-17-92 cluster and its therapeutic potential in dual pathological contexts, cardiovascular diseases, and cancer.

## 1. Introduction 

MicroRNAs (miRNAs) are short non-coding RNAs (ncRNAs), approximately 18–25 nucleotides in length, that serve as vital post-transcriptional regulators via mediation of gene expressions [1, 2, 3]. Recent high throughput sequencing and computational data analyses have uncovered that an individual intracellular miRNA can potentially bind and influence over 100 unique messenger RNAs (mRNAs) simultaneously, exerting pleiotropic effects in various biological processes. miRNAs perform imperfect, complementary base pairing with the three prime untranslated region (3^′^ UTR) of the target mRNA to initiate mRNA cleavage and degradation. This allows the miRNA to successfully repress any further translation of the mRNA, silencing target gene expression, which plays important roles in multiple diverse physiological and pathophysiological processes [4, 5, 6]. miRNAs regulate gene expression involved in numerous physiological processes such as cell growth, proliferation, differentiation, apoptosis and development as well as pathological disorders, including muscular dystrophy, neurological disorders, metabolic diseases, cardiovascular complications, tumorigenesis and malignant development, relating to various tissues and organs [7].

Cardiovascular diseases (CVDs) remain the leading cause of death worldwide, nearly 17.9 million people died from CVD in 2019, nearly 32% of global death (World Health Organization; https://www.who.int/news-room/fact-sheets/detail/cardiovascular-diseases-(cvds). Cancer is the second leading cause of death globally, nearly 10 million deaths in 2020 World Health Organization; https://www.who.int/news-room/fact-sheets/detail/Cancer). An estimated 618,120 people will die from cancer in the United States in 2025 (American Cancer Society; https://www.cancer.org/research/cancer-facts-statistics/all-cancer-facts-figures/2025-cancer-facts-figures.html). Among all miRNAs, a highly conserved cluster, miRNA-17-92 plays numerable roles at the nexus of multiple physiological and pathological processes. It emerges as a crucial regulator during the development of bone, lung, heart and immune system [8], cellular proliferation and differentiation [9], apoptosis [10], angiogenesis [11], normal organ development as well as tumorigenesis [12]. Dysregulation of miRNA-17-92 causes cardiac pathogenesis, related to heart failure, fibrosis, hypertrophy, arrhythmias, atherosclerosis, and coronary artery disease [13, 14, 15]. In addition, miRNA-17-92 plays diverse roles in cancers since it acts as an oncogene in some cancers, whereas it exhibits tumor suppression function in other cancers, depending on the repression of it’s key target genes affecting hall marks of cancer [16]. Attributable to its intricate paradoxical roles, miRNA-17-92 shows promise as a dianostic or prognostic biomarker, which offers it as potential therapeutic target for cardiovascular diseases and cancers. In this review, we provide a comprehensive overview of the mechanistic insight into the multifaceted roles of the miRNA-17-92 cluster in cardiovascular diseases and cancer with hopes of guiding future development of targeted novel RNA therapeutics.

## 2. Biogenesis of miRNAs and Mechanism of Action

The majority of miRNAs are transcribed from intergenic regions of DNA by RNA polymerase II or RNA polymerase III as an initial primary transcript (pri-miRNAs). Pri-miRNAs can be encoded at coding transcripts and even in the exonic regions [17]. Prior to directly modulating the expression of target genes, miRNAs are formed in association with RNA endoribonuclease Dicer and RNA-induced silencing complex (RISC) [18]. RNA polymerase II first transcribes the genes encoding for the miRNAs to form a pri-miRNA resembling a hairpin structure. The primary transcript then undergoes several nuclear and cytoplasmic processing phases to become the final functioning mature miRNA. Specifically, the RNA-binding protein, DiGeorge Syndrome Critical Region Gene 8 (DGCR8), recognizes and binds to the double stranded hairpin loop. This interaction prompts another processing enzyme, Drosha (RNAase III enzyme), to associate with DGCR8, resulting in the formation of a completed micro-processing complex. The microprocessor, composed of Drosha and DGCR8, cleaves the primary miRNA into smaller nucleotide sequences, which are translocated from the nucleus to the cytoplasm by Exportin-5 for further processing. Drosha interacts with the basal junction and UG motif of pri-miRNAs and cleaves to generate pre-miRNAs. DGCR8 helps Drosha to cleave pri-miRNAs precisely and efficiently [19]. Precisely, Rhed (RNA-binding heme domain, amino acids 285–478) of DGCR8 interacts with apical UGU motif of pri-miRNA. Interestingly, three distinct amino acids (461–463) in Rhed of the DGCR8 have the ability to distinguish between UGU- and noUGU of pri-miRNAs, which is critical for miRNA biogenesis.

Dicer, another RNAase III enzyme, recognizes and cleaves the miRNA into a mature, 18–24 nucleotides long, single stranded RNA that is primed for the final cytoplasmic processing. The last step involves further cleavage of the dsRNA (double-stranded RNA) into shortened dsRNA fragments that are assembled by associated proteins onto the active sites of the RNase type III enzyme, RISC [20]. RISC carefully selects and unwinds the strand that will ultimately become the single stranded, final mature product of miRNA (referred as the guide strand) before releasing the non-functional components for degradation [21]. Finally, the single stranded RNA guides RISC to interfere with the transactivation-responsive RNA-binding protein (TRBP) and Argonaute 2 (Ago2), which ultimately inhibits the 3^′^ UTR of the designated target mRNA through complementary base-pair interactions and negatively regulates its expression [22]. The targeted mRNA is either translationally inhibited or completely degraded based on the extent of sequence complementarity between the miRNA and mRNA [23].

In addition to the classical canonical miRNA biogenesis pathway, miRNA may also be synthesized by a non-canonical pathway, known as the mirtron pathway. In this alternate system, short introns are processed by splicing and intron lariat debranching, rather than Drosha/DGCR8 processing [24, 25, 26]. The debranched mirtrons are direct substrates of Exportin-5 and the Dicer-1/loqs system, yielding small RNAs, which require Argonaute RISC component 1 (Ago1) to repress target transcripts [27]. Some mitrons undergo processing via the simtron pathway, which requires Drosha but does not depend on Drosha’s binding partner DGCR8 or the endonuclease Dicer [28]. Despite variations in miRNA biogenesis pathways, all mechanisms produce mature miRNAs that can silence target transcripts via association with the RISC complex, as demonstrated by their interactions with Argonaute proteins.

Almost half of mammalian miRNAs are generated from polycistronic miRNAs (miRNA clusters) [29]. A polycistronic miRNA with multiple components may has enormous capacity for gene regulation, which causes pleiotropic biological effects through coordinated complex mechanisms [30]. One of the best characterized polycistronic miRNAs is miRNA-17-92, which is well conserved in vertebrate species [31].

## 3. Classification and Processing of miRNA-17-92 

**Fig. 1.**
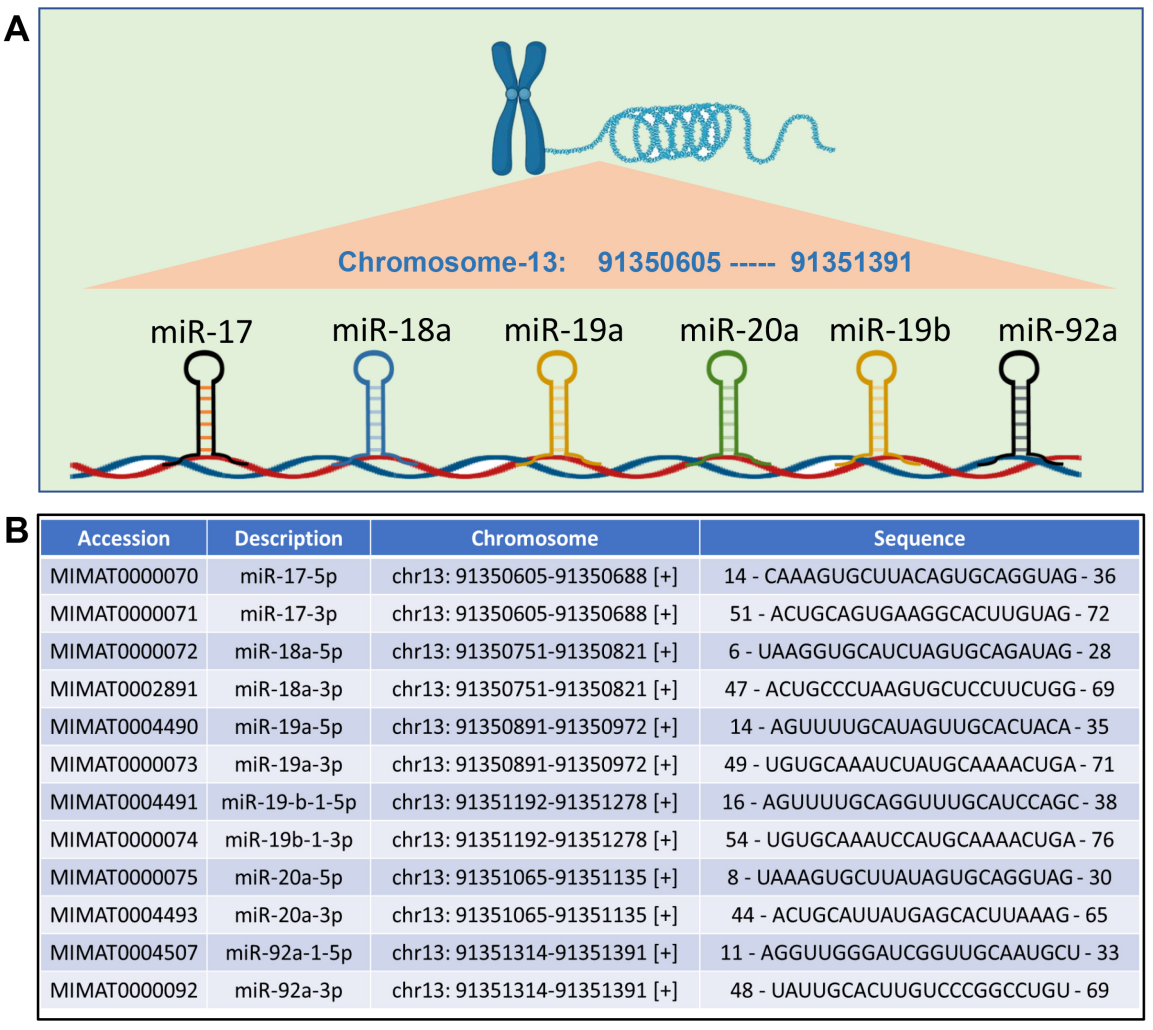
**Schematic representation of miRNA-17-92 family**. (A) The structure of miRNA-17-92 cluster, consisting of miRNA-17, miRNA-18a, miRNA-19a, miRNA-19b, miRNA-20a and miRNA-92a, located on chromosome 13. (B) Details of each member of microRNAs (miRNAs) of miRNA-17-92 cluster (accession numbers, chromosomal locations and sequences). miR, miRNA. Image is created with the help of Biorender.com.

The miRNA-17-92 family, a highly conserved group of miRNAs, was first recognized as an oncogene because of its high serum levels in cancer patients and its abundant expression across various tumor types [32, 33]. Members of this family have been found to promote proliferation and suppress apoptosis of cancer cells with induction of tumor angiogenesis. As a whole, the miRNA-17-92 family consists of three paralogous miRNA clusters which include the miRNA-17-92 cluster, the miRNA-106a-25 cluster and the miRNA-106a-363 cluster. Although these clusters are highly similar with identical 7 mer seed sequences, they reside within different chromosomal locations [32]. The human miRNA-17-92 cluster is composed of seven individual miRNAs (miRNA-17-3p, miRNA-17-5p, miRNA-18a, miRNA-19a, miRNA-19b, miRNA-20a and miRNA-92a), which are located in the third intron of a ~7 kb primary transcript known as C13orf25 (chromosome 13 open reading frame 25) [34] (Fig. 1). The miRNA-106b-25 cluster, which encodes miRNA-106b, miRNA-93, and miRNA-25, is located on human chromosome 7; the miRNA-106a-363 cluster, which encodes miRNA-106a, miRNA-18b, miRNA-20b, miRNA-19b-2, miRNA-92a-2, and miRNA-363, is located on the human X chromosome [32, 35]. Both miRNA-17-92 and miRNA-106b-25 are highly expressed in a wide array of tissues in vertebrates, while miRNA-106a-363 is generally expressed at lower levels [36, 37].

Based on the seed sequences (nucleotides 2–8, the complementary sequences on their target mRNA), the miRNA-17-92 family is also classified into four different sub-families, miRNA-17/106 sub-family (miRNA-17, miRNA-20a/miRNA-20b, miRNA-106a/miRNA-106b, and miRNA-93), miRNA-18 subfamily (miRNA-18a/miRNA-18b), miRNA-19 sub-family (miRNA-19a/miRNA-19b), and miRNA-25/92 sub-family (miRNA-25, miRNA-92a and miRNA-363) [38].

The individual members of miRNA in the miRNA-17-92 are differentially processed to produce mature miRNAs in different tissues [39]. Donayo *et al*. [40] revealed the hierarchy of events in the processing of the miRNA-17-92 polycistron, which orchestrates a wide range of physiological and pathological functions. The miRNA-17-92 cluster adopts a well-defined globular structure where the 5^′^ region of the cluster folds on a 3^′^ core domain that contains the miRNA-19b and miRNA-92 pre-miRNA hairpins and the non-miRNA containing stem-loop (NMSL) [41]. This compact tertiary structure of miRNA-17-92 regulates differential biogenesis of distinct miRNAs within this miRNA cluster due to their accessibility to Drosha. The internalized miRNA-19b and miRNA-92 are processed less efficiently than other miRNAs (miRNA-17, miRNA-19a and miRNA-20a) on the surface of the structure and in close proximity to Drosha [41, 42, 43]. Disruption of the compact tertiary structure exposes Drosha binding domain resulting in increased miRNA-92a expression with increased repression of its target, pro-angiogenic integrinα5 (ITGA5) mRNA [41].

The accessibility of interacting partners to process the enzymes such as Drosha play an important role in the expression level of miRNA-17-92. Accordingly, the miRNA-18a, though is not buried in the 3^′^ core, is also internalized within the globular structure of the cluster and relatively less exposed to Drosha activity compared to miRNA-17, miRNA-19a and miRNA-20a [43]. NMSL sequence is highly conserved between species containing the miRNA-17-92 cluster that are involved in the tertiary structure formation of the cluster. The presence of tandem adenosine repeats in the NMSL of the 3^′^ core and its interaction with 639–641 nucleotides of miRNA-19b, the so called NMSL receptor (NMSLR), plays an important role in the folding of miRNA-17-92 polycistronic cluster [43]. Moreover, disruption of the interaction between NMSL and miRNA-19b also has implication during the maturation process and later in downregulation of the miRNA-92 target ITGA5 [43].

An RNA binding protein (RBP), serine-arginine rich splicing factor 3 (SRSF3) also binds to multiple CNNC (a short, conserved RNA sequence, C-N-N-C, where N is any nucleotide) motifs downstream of Drosha cleavage sites within miRNA-17-92 and promotes the processing of different members of miRNA-17-92 cluster, specifically miRNA-17 and miRNA-20a [44]. SRF3-mediated enhancement of miRNA-17/20 processing alters expression of their target mRNAs encoding key cell cycle inhibitor cyclin-dependent kinase inhibitor 1A (CDKN1A/p21), which leads to enhance cell cycle progression and proliferation in mouse pluripotent cells, human cancer cell lines and primary colorectal tumors [44].

The post-translational modifications of Ago2 play key roles in the biogenesis and processing of miRNAs by regulating Ago2 stability and effective RISC activity [45, 46]. Acetylation of Ago2 specifically increases its binding to the motif UGUGUG in the terminal-loop of pre-miRNA-19b1, which is essential for miRNA-19b biogenesis and proven to promote cancer progression [47].

Despite of dynamic expression profiles and levels, the various expression patterns of different members of miRNA-17-92 still implicate potential functional relationships. Although miRNA-17-92 are transcribed as a polycistronic transcript encoded from a single primary transcript, they show diverse expression pattern. Evolutionarily miRNA-17 and miRNA-19 gene families are conserved and have overlapping distribution pattern across the vertebrates [48]. All members of miRNAs originating from miRNA-17-92 cluster and its two paralogs belongs to four “seed” families namely miRNA-17, miRNA-18, miRNA-19 and miRNA-92. Even though miRNA-17-92 clusters show a remarkable sequences homology within vertebrates, their orthologs are absent in other species. However, miRNA-92 seed family, shows exceptional presence in other non-vertebrate species like *D. melanogaster* and *C. elegans*, implicating their origin at much earlier time point.

## 4. Cardiac Development/Regeneration-Differentiation and Cardiac Aging 

**Fig. 2.**
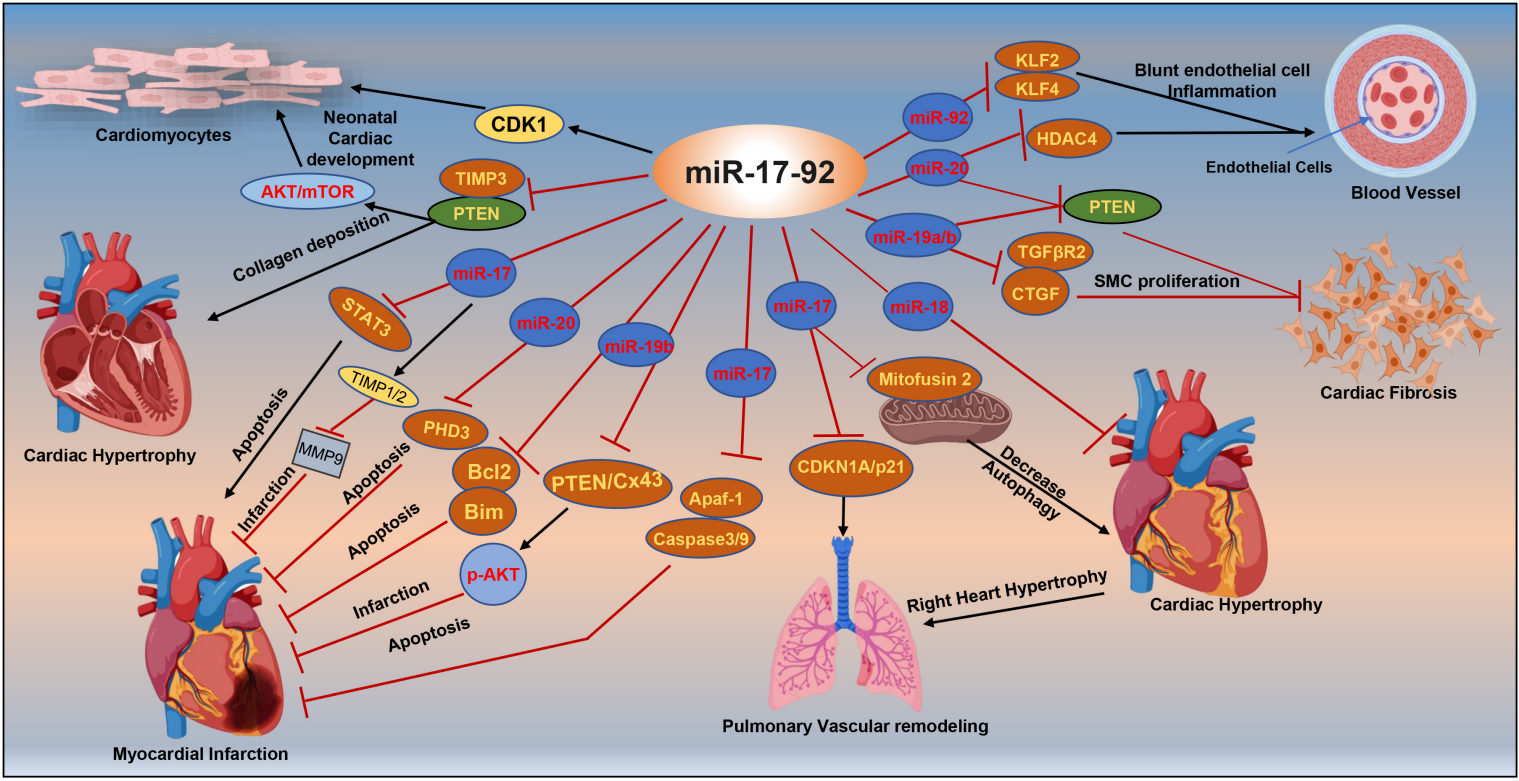
**Targets of miRNA-17-92 associated with cardiac diseases**. mTOR, mammalian target of rapamycin; CDK1, cyclin-dependent kinase 1; PTEN, phosphatase and tensin homolog; STAT3, signal transducer and activator of transcription 3; TIMP3, tissue inhibitor of metalloproteinases 3; MMP9, matrix metalloproteinase 9; Bcl2, B-cell lymphoma 2; CDKN1A/p21, cyclin-dependent kinase inhibitor 1A; KLF2 and KLF4, Krüppel-like factor 2 and 4; HDAC4, histone deacetylase 4; CTGF, connective tissue growth factor; Cx43, connexin43; SMC, smooth muscle cell; TGFβR2, transforming growth factor beta receptor 2; Apaf-1, apoptotic protease activating factor 1; Bim, Bcl-2 interacting mediator of cell death; PHD3, prolyl-hydroxylase 3; AKT, protein kinase B; p-AKT, phospho-AKT; miR, miRNA. Image is created with the help of Biorender.com.

Emerging evidence indicates that the miRNA-17-92 cluster is essential not only in cancer-related processes such as tumorigenesis and metastasis, but also in normal organ development (bone, heart, lung, etc.) as well as in development of immune system, B-cell maturation and differentiation, adipogenesis, in regulation of lipid and carbohydrate metabolism, insulin resistance and bone homeostasis via multiple signaling pathways [49]. The diverse maturity levels of individual members of the miRNA-17-92 cluster were identified in different cell types [50]. For example, the miRNA-17-92 cluster is highly expressed during embryonic development, which allows for regulation of cellular differentiation [36, 51] (Fig. 2). However, concentrations of this cluster’s transcripts decline following terminal differentiation as development proceeds [52]. Germline deletion of the miRNA-17-92 cluster in mice resulted in smaller embryos and immediate postnatal death as a result of lung hypoplasia and ventricular septal defects [36]. Conversely, overexpression of miRNA-17-92 in lung epithelium also promotes the proliferation of epithelial progenitor cells with abnormal lung development, leading to increased mortality following birth [52].

The development of the heart involves the differentiation of cardiac precursors into a cardiac lineage, including the ventricular and atrial myocardium, cardiac mesenchyme, epicardium and endocardium. The miRNA-17-92 cluster plays a crucial role in overseeing cardiac development by regulating differentiation, proliferation, and migration of cardiac stem cells [32, 53]. Bone morphogenetic proteins (BMP) are involved in the regulation of the critical signaling pathways underlying cardiovascular development [54], specifically the differentiation of pre-cardiac mesodermal cells into cardiomyocytes [55]. Several studies indicate a close crosstalk between miRNA-17-92 and BMP signaling [56, 57, 58, 59, 60]. Wang *et al*. [60] identifies a synergistic interaction between BMP-signaling and miRNA-17-92 cluster during early heart development which facilitates myocardial differentiation from cardiac progenitors. BMP-signaling promotes myocardial differentiation from cardiac progenitors by downregulating cardiac progenitor genes via directly regulating Smad-mediated miRNA 17-92 expression. Increased cardiac progenitor genes (*Isl1*, *multipotent Islet1* and *Tbx1*, T-box gene) activity results in defective myocardial differentiation. Using miRNA-17-92 null mice embryos, this study confirms that *Isl1* and *Tbx1* are regulated by miRNA-17-92 [60]. Knockout of the miRNA-17-92 cluster results in insufficient downregulation of cardiac progenitor genes *Isl1* and *Tbx1*, leading to failed differentiation and development of the cardiac outflow tract, right ventricle, and inflow tract. *Isl1* and *Tbx1* expression levels are directly balanced by miRNA-17 and miRNA-20a activity. BMP signaling also negatively regulates *Vegfa* (Vascular endothelial growth factor A) to develop outflow tract development by promoting Smad and miRNA-17-92 [56].

Using cardiac-specific miRNA-17-92 transgenic and knockout mice, Chen *et al*. [53] demonstrates the essential role of the members of the miRNA-17-92 cluster for the induction of cardiomyocyte proliferation in embryonic, postnatal, and adult hearts. Cardiac-specific deletion of miRNA-17-92 mice demonstrated partial embryonic lethality as well as a significant retardation of postnatal cardiac development due to insufficient cardiomyocyte proliferation. Transgenic overexpression of cardiac miRNA-17-92 induces cardiomyocyte proliferation during the neonatal developmental stage [53]. In addition, the members of miRNA-17-92 cluster, specifically miRNA-19, are required to induce neonatal rat cardiomyocyte proliferation via repression of PTEN (phosphatase and tensin homolog), a tumor suppressor [53]. Similarly, overexpression of miRNA-19 in the multipotent murine P19 cell line promotes cellular proliferation, but inhibits serum deprivation-induced apoptosis [61]. miRNA-19b overexpression promotes P19 cell differentiation into mature cardiac cells by inhibiting the activation of WNT (Wingless/Integrated)/β-catenin signaling pathway. Key transcription factors, such as cardiac troponin I (cTnI), GATA binding protein 4 (GATA4), and atrial natriuretic peptide (ANP) are also significantly increased in the miRNA-19b upregulated group when compared to the control group.

Among the different miRNAs that belong in this cluster, several studies showed controversial roles of different members of miRNA-17-92 cluster. Dysregulation of cardiac miRNA-17-92 during cardiovascular morphogenesis leads to a lethal hypertrophic cardiomyopathy and arrhythmogenesis due to unfavorable direct repression of PTEN and connexin43 (Cx43) [14]. Under normal physiological conditions, matured miR20a is downregulated in differentiated cardiomyocytes compared to normal pluripotent stem cells P19 [62]. However, Ai *et al*. [63] demonstrates that overexpression of miRNA-20a in P19 cells represses cell proliferation and differentiation into cardiomyocytes, while enhancing apoptosis with markedly decreased levels of desmin, GATA4, and cardiac troponin T (cTnT). miRNA-20a modulates proliferation, differentiation and apoptosis of P19 cells via direct targeting of Smoothened (SMO), a member of the Hedgehog (Hh) pathway. Overexpression of this miRNA cluster family also contributes to reduced cell proliferation via inhibition of a post-transcriptional protein, Friend of GATA-2 (FOG-2), which is responsible for normal cardiac development [64].

Study with zebrafish embryos reveals that overexpression of miRNA-19b impedes normal heart development through inhibition of the canonical WNT signaling pathway [65]. Compared to the negative control and wild-type groups, transfection of miRNA-19b mimics in zebrafish embryos increases mortality and heart malformation rates in dose dependent manners. Pericardial edema and bradycardia are observed in embryos injected with miRNA-19b mimic, further demonstrating the detrimental effects of miRNA-19b overexpression. In association with cardiac morphology and function, both heart rate and heart structural formation were significantly impaired among miRNA-19b mimic groups.

The cluster also plays a crucial role in the proliferation of cardiomyocytes and vascular endothelial cells. Vascular endothelial growth factor (VEGF) transcriptionally controls all miRNA-17-92 cluster members via the activation of mitogen activated protein kinase (MAPK). In turn, MAPK’s phosphorylation of ETS Like-1 protein (ELK-1) allows for ELK-1’s binding to the miRNA-17-92 promoter sequence and eventual promotion of vascular endothelial cell proliferation [66].

Another member in the family, miRNA-17, was found to slow tissue development by suppressing the expression of fibronectin, an extracellular matrix glycoprotein, and the fibronectin type-III domain containing 3A (FNDC3A) that regulate tissue repair and function [67]. Overexpression of miRNA-17 in Ypen cells (rat endothelial prostate cell lines) increases cell detachment and decreases cell proliferation, adhesion and migration. Transgenic mice expressing miR‑17 show growth retardation rates in the heart, liver, spleen and the entire body following repression of fibronectin and FNDC3A expression.

The miRNA-17-92 cluster has also been identified in playing a crucial role in cardiac aging. Cardiomyocytes are terminally differentiated cells that are unable to further proliferate as the heart ages, leaving the organ especially susceptible to rapid progression of a multitude of diseases. Therefore, therapeutic strategies that minimize the progression of heart failure from senescence will be beneficial. Symptoms of cardiac aging include ventricular wall hypertrophy, loss of elasticity, and aggregation of collagen deposits. Recent research has shown that miRNA-17-92 can target the mammalian target of rapamycin (mTOR) pathway, a cellular pathway that regulates important cell growth processes. Cardioprotective effects were observed when miRNAs inhibited the pro-hypertrophic mTOR pathway [68]. As mice aged during the study, their hearts exhibit reduced miRNA-19a levels. miRNA-19a directly regulates the prostate apoptosis response-4 (PAR-4) pathway that is responsible for apoptosis and cell growth/survival [15].

## 5. The Roles of miRNA-17-92 in Cardiac Diseases

The miRNA-17-92 cluster is expressed in different cardiac cells, including cardiomyocytes, endothelium cells, fibroblasts and cardiac progenitor cells [15]. Dysregulated expression of different members of miRNA-17-92 causes multiple cardiovascular pathogenesis [14, 69].

### 5.1 Myocardial Ischemia/Reperfusion Injury-Myocardial Infarction and Heart Failure

Myocardial infarction (MI) is a pathophysiological condition where blood supply to the myocardium is reduced, resulting in an imbalance between myocardial oxygen supply and oxygen demand [70]. MI can be caused by several factors including narrowed coronary arteries due to atherosclerosis, high metabolic demand with constant coronary flow reserve, and microvascular structural changes that impede blood flow to the heart [71]. The lack of blood flow results in the irreversible death of myocardial cells that compromises the left ventricle function. As a result of MI, cardiomyocytes perish and fail to be replaced as mature cardiomyocytes lack proliferative capabilities.

The most effective therapeutic intervention for limiting MI is acute restoration of blood supply immediately following myocardial ischemia, coined as myocardial reperfusion. However, abrupt restoration of blood supply to the ischemic myocardium paradoxically causes further injury due to hyperactivation of the mitochondria and generation of reactive oxygen species (ROS) [72, 73]. The presence of excessive ROS results in the opening of the mitochondrial permeability transition pore (mPTP) and causes irreversible damage to cardiomyocytes [74]. An incident of myocardial ischemia/reperfusion (I/R) injury activates several cellular signaling mechanisms that result in considerable amount of irreversible loss of cardiomyocyte due to apoptosis, autophagy, pyroptosis and metabolic imbalance [75]. These cellular changes develop lasting consequences in the physiology of the heart leading to cardiac dysfunctions, such as arrhythmias, hypertrophy, dilated cardiomyopathy, cardiac fibrosis and eventually heart failure and death [76].

miRNAs are potent regulators of gene expression in both normal and pathological conditions of the heart and can determine the adverse outcome during a myocardial I/R injury. Several studies indicate complex roles that each member of miRNA-17-92 cluster play in various cardiac cells during myocardial I/R injury (Fig. 2). Further, cell type specific expression of miRNA-17-92 in endothelial, fibroblasts and cardiomyocytes can define the unique function of the miRNA-17-92 cluster in cardiovascular system, especially during the development of pathological conditions such as MI and hypertrophy [77]. Members of the miRNA-17-92 cluster are downregulated in infarct area of heart samples from a murine model of I/R injury [78]. Transgenic overexpression of cardiac miRNA-17-92 protects an adult mouse’s heart against MI-induced injury [53]. Data shows that overexpression of miRNA-17-92 in a mouse’s heart induced the expression of cyclin-dependent kinase 1 in cardiomyocytes, resulting in increased cardiomyocyte proliferation. Moreover, miRNA-17-92 transgenic mice showed decreased scar size and improved cardiac left ventricle function following an acute myocardial infarction [53]. Under diabetic conditions, miRNA-17-92 deficient mice have higher infarct size when compared to wild type mice following myocardial I/R injury [79]. This work identifies the interplay between miRNA-17-92 with PHD3 (prolyl hydroxylase 3)-protein kinase B (AKT) signaling in post-I/R diabetic mice, continuing to highlight the potential for therapeutic applications of this cluster [79].

Members of the miRNA-17-92 cluster possess diverse roles within the cardiovascular system, largely due to their expression in multiple cell types. The expression of specific member of miRNA-17-92 in an individual cell type can have profound influence on the outcome against I/R injury. Low circulating miRNA-19a expression was associated with high mortality risk in patients with coronary artery disease [80]. Anti-apoptotic properties of miRNA-19a/b against myocardial I/R injury have been reported in several *in vivo* and *in vitro* experimental studies [78, 81]. Specifically, miRNA-19 was demonstrated to play a role in cardiomyocyte proliferation by targeting PTEN [53]. The expression level of PTEN and Cx43, two targets of miRNA-19a/b, are suppressed in hearts with a concomitant increase of phosphorylated AKT (Ser473), a downstream signaling member of PTEN. Overexpression of miRNA-19b reduces the infarct area in mice hearts with an increase in the left ventricular systolic function and a decrease in cardiomyocyte apoptosis by targeting the pro-apoptotic gene Bcl2 l11/Bim [78, 81]. Interesting study from Gao *et al*. [82] shows that systemic intra-cardiac administration of miR19a/19b mimics in adult mice enhances cardiomyocyte proliferation and regeneration of cardiac cells in response to MI. They reveal that miRNA-19a/19b reduces myocardial infarction with improvement of cardiac function which in turn increases survival rates. The miRNA-19a/19b substantially reduces both cardiomyocytes and non-cardiomyocytes apoptosis via down regulation of Bim and PTEN expression in hearts. By directly targeting PTEN, miRNA-19b activates AKT signaling to protects cardiomyocytes from H_2_O_2_-induced apoptosis [83]. miRNA-19a/19b also reduces immune response by a reduction of M1-type inflammatory macrophages [82]. Delivering miRNA-19a/b by a novel supramolecular hydrogel for sensitive scavenging of ROS in rats and mini pigs, a recent study demonstrates that controlled release of miRNA-19a/b induces cardiomyocyte proliferation in infarcted hearts with improving cardiac function by alleviating oxidant stress, inflammation, fibrosis and apoptosis [84]. The study also reveals that miRNA-19a/b promotes cardiomyocyte proliferation through phosphatidylinositol 3-kinase (PI3K)/AKT signaling pathway.

Numerous studies reveal the controversial roles of miRNA-17-5p, another member of miRNA-17-92 family, in protection against MI [85, 86, 87, 88, 89, 90, 91, 92, 93]. Similarly, overexpression of miRNA-17-5p increases apoptosis in cardiomyocytes by targeting signal transducer and activator of transcription 3 (STAT3) and inhibition of miRNA-17-5p blunts I/R injury in mice [87]. Pre-treatment with locked nucleic acid (LNA)-antimiRNA-17-5p two days prior to I/R injury in mice via tail vein injection reduces infarct size and blocks apoptosis [87]. Inhibition of endogenous miRNA-17 via administration of antagomir in mice enhances expression of tissue inhibitors of metalloproteinases 1 and 2 (TIMP1 and TIMP2) with reduction of matrix metalloproteinase 9 (MMP9) activity, which leads to reduced post-MI infarct size and cardiac dysfunction [88]. Inhibition of miRNA-17-5p alleviated myocardial ischemia/reperfusion (MI/R)-induced cardiac injury by reducing cardiomyocyte apoptosis, myocardial autophagy, remodeling and fibrosis via targeting STAT3 and regulating endoplasmic reticulum (ER) stress [85, 93]. Administration of miRNA-17 antagomir in infarcted myocardium of streptozotocin (STZ)-induced diabetic mice prevents impairment of angiogenesis and improved cardiac function [91].

Conversely, several other studies report beneficial role of miRNA-17 against myocardial infarction [79, 86, 89, 90, 92]. Exogenous delivery of miRNA-17 in cardiomyocytes suppresses Apaf-1 expression, which consequently attenuates the formation of the apoptosome complex by inhibiting the cleavage of procaspase 9 and caspase-3 activation, which prevented norepinephrine-induced apoptosis [90]. Our study has noticed that rapamycin provides cardio protection against MI/R injury by promoting induction of miRNA-17 and miRNA-20. These two miRNAs repress the pro-apoptotic prolyl hydroxylase (Egl-9 family hypoxia inducible factor 3 (Egln3)/PHD3) expression in diabetic mice [79]. Rosuvastatin (an β-hydroxy β-methylglutaryl-(HMG)-CoA reductase inhibitor) is also shown to attenuate MI/R injury via promotion of miRNA-17-3p-mediated autophagy and inhibition of cardiomyocyte apoptosis [94]. Shi *et al*. [95] reveals that therapeutic application of miRNA-17-3p agomir promotes exercise-mediated cardiomyocyte proliferation by targeting tissue inhibitor of metalloproteinases 3 (TIMP3) and PTEN-AKT pathway, effectively providing cardio protection against MI/R injury.

Exosomes, a subtype of extracellular vesicles, regulate different gene expressions in target organs by delivering bioactive substances such as proteins, lipids, DNAs, mRNAs, miRNAs, long non-coding RNA (lncRNA) and circular RNAs [96]. Exosomal miRNAs are widely involved in the occurrence and development of cardiovascular diseases, and are expected to be an attractive therapeutic tool in the diagnosis, treatment, and prevention of cardiovascular diseases. Expression of miRNA-17-3p is notably decreased in the peripheral blood exosomes in patients with cardiac I/R injury when compared to healthy individuals [89]. Exosomes are used as an efficient drug delivery vehicle due to their low toxicity, minimal immunogenicity and its high biocompatibility and their ability of resist biological barriers [97]. Delivering miRNAs via exosomes is an attractive therapeutic strategy for heart failure (HF) treatment. Therefore, engineered miRNA-17-92 cluster–enriched exosomes are used for therapeutic purpose and are shown as efficient carriers compared to traditional liposomes mediate delivery system [98, 99, 100]. Mesenchymal cell derived exosome loaded with miRNA-17-92 promote more endogenous myocardium and improve post-myocardial infarction cardiac repair [101]. Intravenous administration of exosomal miRNA-17-3p before myocardial I/R injury in mice alleviates cardiac injury by targeting TIMP3 and inhibiting myocardial inflammatory cell infiltration, fibrosis, and necrosis [89]. Delivery of engineered Tβ4-exosome (Tβ4-EXO, thymosin β4 is a key pro-angiogenic factor) in mice stimulates the formation of collateral circulation after myocardial infarction and enhances the angiogenesis of coronary artery endothelial cells via the miRNA-17-5p/PHD3/HIF-1α pathway [86]. Tβ4-exosome induces the expression of exosomal-miRNA-17-5p, which inhibits ubiquitination of HIF-1α by targeting PHD3. The suppression of miRNA-17-5p drastically abrogates the stimulation of angiogenesis, as well as the expression of angiogenic-related genes, which in turn supports the notion that miRNA-17-5p improves Tβ4-EXO-mediated myocardial angiogenesis and cardiac function following myocardial I/R injury [86].

Another member of the miRNA-17-92 cluster, miRNA-20a, is shown to inhibit apoptosis in post-hypoxic neonatal rat cardiomyocytes (NRCMs) [102]. Reports suggest that miRNA-20a targets the pro-apoptotic prolyl hydroxylase Egln3/PHD3, which evokes programmed cell death in cardiomyocytes. Moreover, adenovirus mediated overexpression of miRNA-20a *in vivo* inhibits hypoxia-induced apoptosis [102]. Following hypoxia and reoxygenation (H/R), miRNA-20a level are diminished in rat myoblast cells and H9c2 cells [103]. The miRNA-20a mimics facilitate cardiomyocyte viability and reduce H/R-triggered cardiomyocyte apoptosis by targeting TLR4 (toll-like receptor 4)-mediated inflammatory responses and repressing p38 MAPK/c-Jun N-terminal kinase (JNK) signaling. Conversely, the miRNA-20a inhibitors further induce post-H/R activation of p38/MAPK/JNK signaling in H9c2 cells [103, 104]. Patients with coronary artery disease, when compared to healthy counter parts, display reduced expression of miRNA-20 and increased upregulation of VEGF and PTEN in both their plasma and within coronary artery endothelial cells. Exercise-induced increases in miRNA-20 concentrations also shows suppression of PTEN mediated signaling pathways and ameliorates coronary vascular disorder [105]. Overexpression of miRNA-20a-5p also alleviates oxidized low-density lipoprotein (oxLDL)-induced inflammation and injury by targeting histone deacetylase 4 (HDAC4) in human coronary artery endothelial cells, showing promise in coronary artery disease (CAD) progression prevention [104]. The Framingham Heart Study (FHS) which involved patients with HF, reports that lower abundance of circulating miRNA-17-5p and miRNA-20a-5p in plasma were associated with higher incidences of all cause of HF [106]. By enrichment analysis, the study identifies that miRNA-17 and miRNA-20 target a set of genes involved in the specified pathways relevant to HF (e.g., transforming growth factor-β signaling, growth/cell cycle, and apoptosis) and pathways relevant to myocardial remodeling in a large community-based sample [106].

In contrast to other members of miRNA-17-92, several studies reveal the involvement of miRNA-92a in the development of several cardiac diseases, such as endothelial inflammation, atherosclerosis, and I/R injury [107, 108]. In mouse models of AMI, elevated level of miRNA-92a block angiogenesis by targeting several proangiogenic proteins, including the integrin subunit alpha 5 [109]. Systemic administration of an miRNA-92a inhibiting antagomir improves post-MI neovascularization, promotes cardiac functional recovery, and reduces myocardial infarct size. Enhanced level of miRNA-92a by oxLDL in endothelial cells and in atheroprone areas of hypercholesterolemic mice (LDL receptor, *Ldlr* KO mice) promoted endothelial activation and the development of atherosclerotic lesions. Inhibition of miRNA-92a with antagomiR in these mice protected against endothelial inflammation and dysfunction with reduction of atherosclerotic lesions [108]. The miRNA-92a inhibition results in increased expression of the key endothelial transcription factors, KLF2 and KLF4 (Krüppel-like factor 2 and 4), which are associated with reduction in activated NFκB and endothelial dysfunction and inflammation. Using a porcine myocardial infarction model, Hinkel *et al*. [107] demonstrates that inhibition of miRNA-92a via local catheter-based delivery of an LNA-based miRNA-92a inhibitor significantly improves cardiac functional recovery with reduction of infarct size following MI/R injury. Direct inhibition of miRNA-92a in endothelial cells or directly in inflammatory cells also reduces cell adhesion [107]. Inhibition of miRNA-92a also protects cardiomyocytes against H/R-induced cell death and apoptosis [107]. They also show that a global deletion of miRNA-92a preserves cardiac function to a larger extent than cardiomyocyte-specific miRNA-92 deletion post AMI, suggesting the critical contribution of miRNA-92a in large multitude of cell types including endothelial cells, inflammatory cells, and cardiomyocytes. Similarly, using a porcine reperfused AMI model, another study reports that early single intracoronary administration of encapsulated antagomiRNA-92a prevents left ventricular remodeling without any adverse effects within infarct territories [110].

The miRNA-92a also regulates endothelial cell autophagy by suppressing autophagy-related genes and cardiomyocyte metabolism by repressing transporter proteins [111]. Inhibition of miRNA-92a with LNA-92a (locked nucleic acid based antimiRNA-92a) in mice enhances endothelial autophagy by upregulating Atg4 and promoting revascularization into damaged areas, which leads to cardiac preservation following a MI [111]. In neonatal rat cardiomyocytes, LNA-92a restores metabolism regulated genes, specifically the high-density lipoprotein transporter Abca8b (ATP-binding cassette, subfamily A, member 8b) and the fatty acid translocase cluster of differentiation 36 (CD36), allowing for increased fatty acid uptake and overall improvement in mitochondrial function [111].

There is compelling clinical evidence that elevated levels of miRNA-92a in blood samples may be a potential prognostic biomarker of the patients with AMI and HF [112, 113]. Among older diabetic patients diagnosed with HFpEF (heart failure with preserved ejection fraction), circulating miRNA-92 are significantly upregulated when compared to healthy controls [114]. Treatment with Empagliflozin, an SGLT2 (sodium glucose cotransporter 2) inhibitor, improves endothelial function by repressing miRNA-92a in frail HFpEF patients with diabetes [114]. Evidently, miRNA-92 may serve as a potential therapeutic target for patients with myocardial I/R injury and HF.

### 5.2 Cardiac Fibrosis

After AMI, activated cardiac fibroblasts initially participate in the tissue repair and healing process, which is beneficial for the preservation of tissue homeostasis to prevent ventricular wall rupture [115]. However, overactive cardiac fibrosis due to the deposition of extracellular matrix (ECM) proteins and formation of collagen type I-containing scars causes wall and septal stiffening and progressively worsening cardiac function (Fig. 2). Subsequently, this leads to pathogenic ventricular remodeling which is the key factor in the development of heart failure following MI [116]. Several miRNAs have been demonstrated to regulate cardiac fibrosis by targeting key genes associated with anti-fibrotic or profibrotic signaling pathways [117]. Specifically, miRNA-17-92 plays a critical role in age-related cardiac remodeling and HF by regulating the ECM proteins, connective tissue growth factor (CTGF) and thrombospondin-1 (TSP-1) [69]. Increased levels of CTGF and TSP-1 correlate with TGFβ (transforming growth factor-β) induction, activation of MMPs (matrix metalloproteinases), excessive cardiac fibrosis, and left ventricular stiffening [118, 119]. In the aging-associated HF mice model, profibrotic proteins CTGF and TSP-1 were increased in the hearts with reduction of the expression of all members of miRNA-17-92 cluster as compared to their young littermates. *In vitro* studies with the aged cardiomyocytes and cardiac fibroblasts showed that mimics of miRNA-18a and miRNA-19b blunted the induction of the expression of CTGF and TSP-1, while the inhibition of these miRNAs enhanced CTGF and TSP-1 levels. Similarly, among the members of miRNA-17-92 cluster, miRNA-18a, miRNA-19a and miRNA-19b, were significantly reduced in the cardiac biopsies of idiopathic cardiomyopathy (ICM) patients in old age with HF, with concurrent induction of the transcript levels of CTGF and TSP-1 [69].

The miRNA-19 also regulates cardiac fibroblast proliferation and migration by targeting PTEN, which inhibits the PI3K/AKT signaling [120]. The expression of miRNA-19a-3p/19b-3p is noticeably reduced in the plasma samples of patients with dilated cardiomyopathy, especially in the end stage of dilated cardiomyopathy [121]. Overexpression of miRNA-19a-3p/19b-3p inhibits interstitial fibrosis, epithelial mesenchymal transition (EMT), ECM production and invasion of human cardiac fibroblasts (HCF) [121]. By directly targeting TGFβR2 (TGFβ receptor 2), miRNA-19a/b negatively regulates TGFβ1/Smad2 signaling and inhibits autophagy mediated fibrogenesis in HCF [121]. A similar anti-fibrotic benefit of miRNA-19 was established in rat hearts following MI and angiotensin II (Ang II)-treatment [122]. The expression of miRNA-19 was reduced in the rat hearts following MI and Ang II-treatment, as well as in Ang II-treated cardiac fibroblasts with increased levels of collagen I/II and TGFβ. Overexpression of miRNA-19 alleviates post-MI and Ang II-induced cardiac fibrosis via targeting CTGF and attenuating the MAPKs pathway.

In a rat model with streptozotocin-induced diabetic cardiomyopathy, overexpression of miRNA-20a-5p decreased accumulation of collagen I and TGFβ 1 levels as well as reduced the levels of proinflammatory cytokines (interleukin-6, tumor necrosis factor-α and IL-1β), which mitigate cardiac fibrosis and inflammation [123]. The specific role of miRNA-92a in the cardiac fibrosis process is still controversial. There is a correlation between elevated levels of miRNA-92a and enhanced WNT/β-catenin signaling in exosomes from cardio sphere-derived cells (CDCs). These miRNA-92a enriched exosomes activate bone morphogenetic protein signaling in cardiomyocytes, promote myocyte contractility, improve cardiac function and prevent cardiac fibrosis in a mouse model of AMI [124].

### 5.3 Hypertrophy

Following physiological and pathological stimuli, the heart initially develops hypertrophy to increase contractility and reduce ventricular wall stress as an adaptive response, however, prolonged cardiac hypertrophy eventually results in impaired cardiomyocyte viability, contractile dysfunction, and heart failure [125, 126]. Multiple studies highlight the importance of miRNA-17-92 in the development of cardiac hypertrophy following different stimuli (Fig. 2). Danielson *et al*. [14] reveal uncontrolled expression of miRNA-17-92 during cardiovascular morphogenesis results in lethal cardiomyopathy. In murine model, constitutively overexpression of miRNA-17-92 in the developing cardiovascular system causes a lethal hypertrophic and dilated cardiomyopathy due to the repression of PTEN and Cx43, lead to sudden cardiac death [14].

Pro-hypertrophic role of miRNA-19a/b is identified by overexpressing miRNA-19a/b in neonatal rat cardiomyocytes [127]. Directly targeting the anti-hypertrophic genes atrogin-1 and MuRF-1 (muscle RING-finger protein-1), miRNA-19a/b induces cardiomyocyte hypertrophy with inducing hypertrophic markers. Pro-hypertrophic calcineurin/NFAT (nuclear factor of activated T-cells) signaling has been elevated markedly in cardiomyocytes after overexpression of miRNA-19b. However, miRNA-19b prevents ER-stress-induced cardiomyocyte apoptosis via up-regulation of NFAT target gene encoding α-crystallin-B and repression of the pro-apoptotic gene Bim (Bcl-2-interacting mediator of cell death) [127]. Another study by Lai *et al*. [128] reports that one of the lncRNA, HOX transcript antisense intergenic RNA (HOTAIR) is competing with miRNA-19, thereby regulating PTEN expression and inhibiting the progression of hypertrophic growth of cardiomyocytes induced by Ang-II treatment.

The miRNA-17-5p is also associated with pathological cardiac hypertrophy [129]. An abnormally high expression of miRNA-17-5p has been identified in the heart of transverse aortic constriction (TAC)-induced cardiac hypertrophic rats and Ang II-induced hypertrophic neonatal rat ventricular myocytes (NRVMs), which targets mitochondrial fusion protein mitofusin 2 (Mfn2)-mediated PI3K/AKT/mTOR pathway and suppresses autophagy to promote cardiac hypertrophy [129]. Moreover, the inhibitor of miRNA-17 also improves heart and lung function in chronic hypoxia-induced pulmonary hypertrophic mice by interfering with pulmonary vascular remodeling and right ventricular hypertrophy via up-regulating its target, the cyclin-dependent kinase inhibitor 1A (p21) [130].

The miRNA array analysis demonstrates an increased expression level of miRNA-20 in myocardium of patients with hypertrophy compared with normal myocardium [131]. miRNA-20 is also highly expressed in Ang II-induced hypertrophic cardiomyocyte with reduced expression of Mfn2. Inhibition of miRNA-20 expression rescues Mfn2 expression at transcriptional and translational levels and attenuated Ang II-induced cardiomyocyte hypertrophy.

Conversely, a beneficial role of miRNA-18 has been identified in hypertension-induced cardiac hypertrophy in spontaneous rat hypertensive model and Ang-II treated neonatal rat ventricular cardiomyocytes [129]. Loss of miRNA-18 in the heart severely impairs cardiac functions by triggering heat shock transcription factor 2 (HSF2) and IGF-IIR (insulin growth factor receptor II), which leads to induce cardiac hypertrophy during hypertension. Restoration of cardiac-specific miRNA-18 expression in spontaneously hypertensive rats alleviates the hypertension-induced cardiac dysfunction. Similarly, overexpression of miRNA-18 also represses phenylephrine-induced neonatal rat cardiomyocyte hypertrophy [132].

Using a mouse exercise model, Shi *et al*. [95] demonstrate the advantageous role of miRNA-17-3p in exercise-induced physiological cardiac hypertrophy and in protection against adverse remodeling following myocardial I/R injury. The study shows that miRNA-17-3p in heart of exercise mice is upregulated, but it is decreased in TAC-induced pathological hypertrophy as well as human diabetic cardiac samples. Induction of miRNA-17-3p regulates TIMP3 and PTEN/AKT in the heart of exercised mice, which leads to enhanced proliferation of cardiomyocytes via activating epidermal growth factor receptor (EGFR)/JNK/specificity protein 1 (SP-1) signaling. Overexpression of miRNA-17-3p with agomir following I/R injury preserves cardiac function by reducing cardiac apoptosis and fibrosis. Following I/R injury, mice treated with agomir of miRNA-17-3p increases Ki-67-positive cardiomyocytes, indicating enhanced cardiomyocyte proliferation [95]. The study also confirms that serum miRNA-17-3p level is increased after exercise in mice as well as in heart failure patients.

### 5.4 Angiogenesis 

**Fig. 3.**
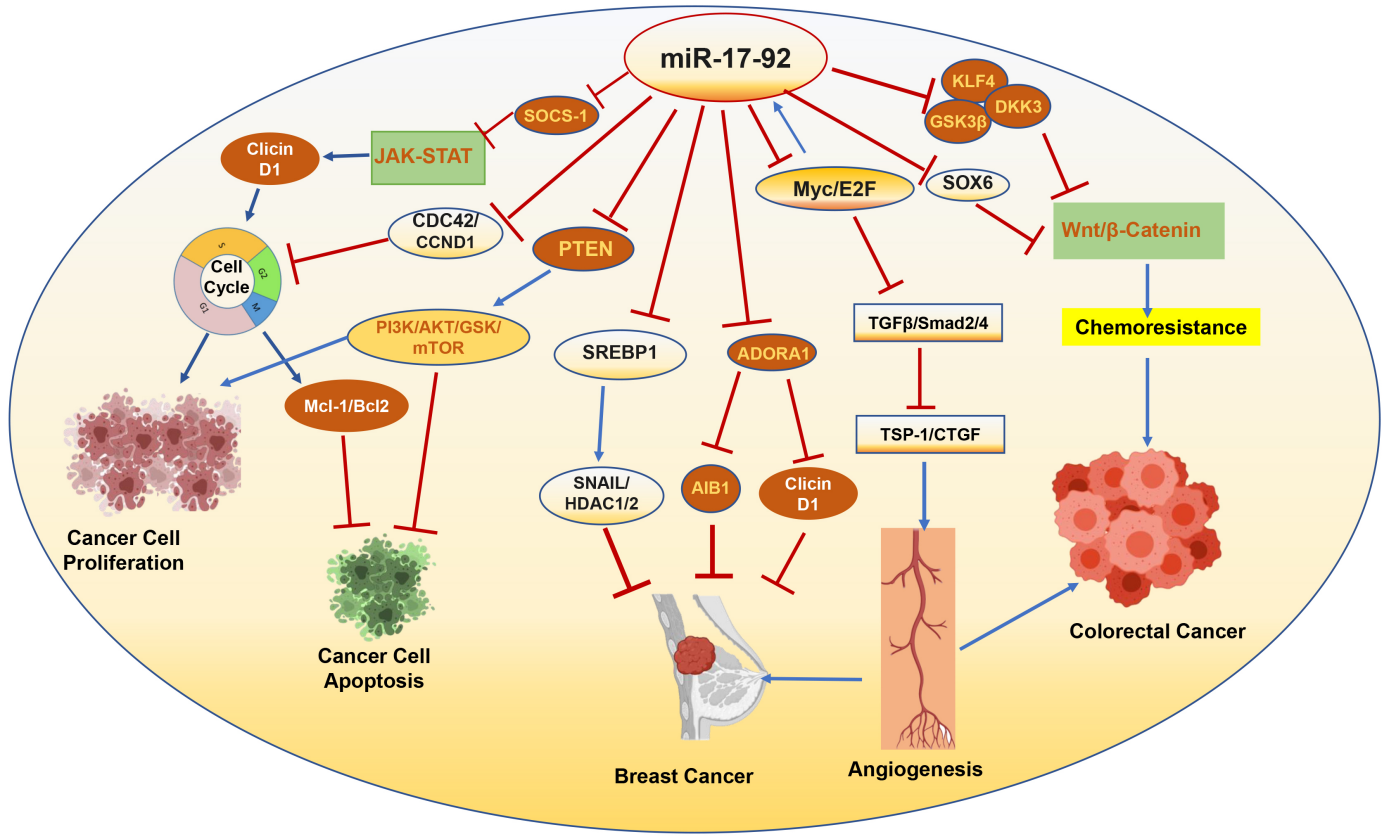
**Targets of miRNA-17-92 associated with progression or suppression of cancer**. SOCS-1, suppressor of cytokine signaling-1; JAK-STAT, Janus kinase/signal transducer and activator of transcription; CDC42, cell division cycle 42; CCND1, cyclin D1; GSK, glycogen synthase kinase; Mcl-1, myeloid cell leukemia 1; SREBP1, sterol regulatory element binding transcription protein 1; ADORA1, adenosine A1 receptor; AIB1, amplified in breast cancer 1; TGFβ, transforming growth factor β; TSP-1, thrombospondin-1; KLF4, Krüppel-like factor 4; DKK3, Dickkopf-3; PI3K, phosphoinositide 3-kinase; SNAIL, zinc finger protein SNAI1; HDAC1/2, histone deacetylase 1/2; Myc, myelocytoma; E2F, early region 2 binding factor; Smad2/4, suppressor of mothers against decapentaplegic 2/4; SOX, SRY-related HMG-box transcription factors; HMG, β-hydroxy β-methylglutaryl; TSP-1, thrombospondin-1; miR, miRNA. Image is created with the help of Biorender.com.

The role of angiogenesis in the prognosis of and protection against cardiovascular disease has the potential to be a promising target for medical therapy. The division and proliferation of existing endothelial cells (EC) help to restore adequate blood-oxygen supply and minimize necrosis following an ischemia/reperfusion injury. Studies have demonstrated that miRNA-17-92 cluster expression is required for both developmental angiogenesis as well as angiogenesis during adulthood [32, 37, 133] (Fig. 2). The miRNA-17-92 cluster was among the first miRNAs known for augmenting tumor angiogenesis [134] (Fig. 3). Elevated levels of c-Myc oncoprotein induce neovascularization by stimulating the expression of the miRNA-17-92 cluster [135]. The proangiogenic role of miRNA-17-92 has been attributed to the repression of the antiangiogenic proteins, TSP-1, and CTGF [134].

The expression of miRNA-17-92 cluster in endothelial cells is positively regulated by VEGF/ERK/ELK-1, the major proangiogenic signaling proteins that modulate blood vessel formation [133]. Endothelial postnatal inactivation of miRNA-17-92 cluster causes impairment of retinal, ear, and tumor angiogenesis [133]. Studies show that miRNA-17-5p, miRNA-18a, and miRNA-19a act as proangiogenic agents by regulating JAK1, while miRNA-92a represses angiogenesis through the regulation of integrin subunit alpha5 [77, 109, 136].

In contrast, the antiangiogenic activity of different members of miRNA-17-92 cluster has been also identified in ECs [77]. Doebele *et al*. [77] reveal that overexpression of the individual members of the miRNA-17-92 cluster, miRNA-17, miRNA-18a, miRNA-19a, and miRNA-20a, reduced EC sprouting, whereas inhibitors of these miRNAs, specifically miRNA-17 and miRNA-20a, augment angiogenesis. In HUVECs, overexpression of histone deacetylase 9 (HDAC9) enhances endothelial cell sprouting by repressing of miRNA-17-92 cluster and controlling the expression of angiogenesis-relevant genes, JAK1 [137]. Silencing of HDAC9 in ECs increases the expression of miRNA-17-92. Inhibition of miRNA-17-20a completely rescues the impaired angiogenic sprouting induced by HDAC9 silencing and blocking miRNA-17 expression also partially reverses the sprouting defect induced by HDAC9 depletion.

Similarly, miRNA-17-3p inhibits intrinsic angiogenesis in ECs by downregulating the cell growth signaling pathways necessary for angiogenesis, such as suppressing Flk-1, a receptor for VEGF [138]. In fact, overexpression of miRNA-17-3p in HUVEC cells reduces the levels of Flk-1, implicating Flk-1 as the primary target of miRNA-17-3p. The anti-angiogenic mechanisms of miRNA-17-3p reduced the cellular growth by modulating the interacting proteins, such as AKT and cyclin D [138]. In STZ-induced diabetic mice, miRNA-17 was upregulated, while VEGFA was downregulated following MI [91]. Inhibition of miRNA-17 improved angiogenesis within the infarct myocardium of diabetic mice with improved cardiac function and reduced infarct size.

Increasing evidence shows that angiogenesis is enhanced by antagomirs that target miRNA-92a. Promoting angiogenesis is achieved by inhibition of miRNA-92 after both induced myocardial infarction and damage to carotid arteries in rats through upregulating endothelial proteins, mitogen-activated protein kinase kinase 4 (MKK4) and KLF4 [139]. Treatment with ant-miRNA-92a also enhances endothelial nitric oxide (NO)-synthase (eNOS) expression in endothelial cells and increases the bioavailability of NO [139]. In mouse models of limb ischemia and MI, systemic administration of an antagomir of miRNA-92a enhances blood vessel growth and recovers cardiac function by stimulating the secretion of pro-angiogenic factors, including integrin subunit alpha5 [109]. The mRNA expression of eNOS is also suppressed in HUVEC cells overexpressing miRNA-92a, which controls vascular tone and is essential for postnatal neovascularization [109]. Moreover, endothelial-specific miRNA-17-92 KO mice exhibits accelerated blood flow recovery and enhances arterial vessel density after limb ischemia [140]. Specifically, miRNA-19a down-regulates WNT-related receptors, Frizzled receptor 4 (FZD4) and the low-density lipoprotein receptor-related protein 6 (LRP6), and β-catenin–dependent gene expression [140]. Antagonism of miRNA-19a/b in aged mice improves blood flow recovery after ischemia and augments WNT signaling by enhancing tissue levels of FZD4 and LRP6 [140].

### 5.5 Arrhythmia

Alteration of cardiac electrophysiological properties and underlying ion channel dysfunction cause abnormalities in ventricular action potential duration that leads to arrhythmias and sudden cardiac death [141]. Studies reveal that miRNA-17-92 is playing critical role in development of cardiac arrhythmia by regulating cardiac electrophysiological homeostasis with modulating electrical conduction and ion channel function [14, 142, 143]. After ischemia, the reduced expression of the gap junction protein Cx43 reduces electrical coupling and accelerates the incident of ventricular arrhythmias [144]. Overexpression of the miRNA-17-92 cluster in cardiomyocytes leads to lethal hypertrophic and spontaneous cardiac arrhythmia, partially by suppressing PTEN and Cx43 [14]. Specifically, the reporter assays confirm that miRNA-19a/b directly targets Cx43 [14]. Bioinformatics with predicted target analysis with hypothermic I/R arrhythmic rat heart suggests that aberrantly expressed miRNA-17 and miRNA-19 may regulate cardiac electrophysiological homeostasis by modulating electrical conduction and ion channel function [145] via targeting *GJA1* gene. The normal expression of gap junction protein Cx43 coded by gene GJA1 is crucial for electric coupling and conduction between cardiomyocytes [146].

In contrary, Wang *et al*. [143] demonstrates the beneficial role of miRNA-17-92 against atrial fibrillation. Paired-like homeodomain transcription factor 2 (Pitx2) is highly expressed in multiple sites of myocardium to regulate cardiac electrical activity by repressing the sinoartrial node program, causes atrial fibrillation, the most common sustained cardiac arrhythmia [147]. An integrated genomic approach identifies that Pitx2 positively regulates miRNA-17-92 and miRNA-106b-25 [143]. Mice with miRNA-17-92 and miRNA-106b-25 deficiency are susceptible to pacing-induced atrial fibrillation compared with wild-type mice. They prove that miRNA-17-92 and miRNA-106b-25 directly repress genes, such as *Shox2* and *Tbx3*, that are required for sinoatrial node development. Similarly, miRNA-19b deficient zebrafish exhibits prolonged ventricular action potential duration caused by impaired repolarization [142]. miRNA-19b directly and indirectly regulates cardiac ion channel expression, specifically the voltage-gated potassium channel potassium voltage-gated channel subfamily E member 4 (KCNE4), to cause action potential prolongation [142].

### 5.6 Diabetes-Related Cardiovascular Complications

Cardiovascular disease remains the leading cause of morbidity and mortality in patients with diabetes. Diabetes mellitus (DM) escalates myocardial susceptibility to I/R injury with poor prognosis and premature morbidity and mortality [148, 149, 150]. In addition, different cardiac structural and functional changes in the patients with diabetes have established diabetic cardiomyopathy, which result in oxidative stress, hypertrophy, fibrosis, cardiac diastolic dysfunction and eventually systolic heart failure [151]. Heart failure as a result of DM is associated with hyperglycemia, which leads to hyperinsulinemia with increased systemic stress and inflammatory response [152]. Studies establish that miRNA-17-92 cluster is involved in neonatal islet β-cell differentiation and development as well as postnatal β-cell maturity to maintain glucose-stimulated insulin secretion (GSIS) [153, 154]. Conditional knockout of miRNA-17-92 cluster in mouse pancreatic β-cells impairs glucose tolerance and promotes diabetes, possibly mediated by PTEN/AKT signaling pathway [155].

Critical roles of independent members of miRNA-17-92 in regulation of signaling pathways of glucose metabolism have been established. By targeting Menine, a negative regulator of β-cell proliferation, miRNA-17 regulates glucose homeostasis in diabetic mice [156]. In hepatocytes, miRNA-19a activates glycogenesis and AKT/ glycogen synthase kinase (GSK) pathway via down-regulating PTEN expression [157]. Li *et al*. [158] reveals that plasma miRNA-19a expression is significantly decreased in diabetic patients accompanied by increased expression of suppressor of cytokine signaling (SOCS)-3, which leads β cell dysfunction to promote the development of diabetes.

A persistent activation of mTOR signaling is associated with diabetic-associated cardiovascular complications [159, 160, 161]. Enhanced mTOR activation impairs cardiac insulin metabolic signaling, which induces serine phosphorylation, but reduces tyrosine phosphorylation of IRS-1/2 (insulin receptor substrates-1/2) and attenuates PI3K-AKT/eNOS activation and NO production [161]. Our studies reveal that inhibition of mTOR with rapamycin treatment improves cardiac function in diabetic mice by attenuating oxidative stress and modulating glucose metabolic proteins as well as protecting hearts against myocardial I/R injury [159, 160, 162]. Rapamycin increases phosphorylation of STAT3 and miRNA-17/20a expression, but suppresses the pro-apoptotic protein prolyl hydroxylase (Egln3/PHD3, a target of miRNA-17/20a) in diabetic mice hearts following I/R injury [79]. The infarct-limiting effect of rapamycin has been abolished in cardiac-specific miRNA-17-92-deficient diabetic mice, which indicates that induction of the STAT3-miRNA-17-92 signaling axis plays a critical role in attenuating MI in rapamycin-treated diabetic mice.

Rising evidence specifies that dysregulated different members of miRNA-17-92 cluster in plasma or hearts play key roles in diabetic cardiomyopathy development [49, 163], which could be used as discerning biomarkers for early diagnosis of diabetic complications or as potential therapeutic targets for patients with diabetes.

Collectively, accumulating evidence has confirmed the diverse roles of different members of miRNA-17-92 in multiple cardiac diseases with associated molecular mechanisms, which advances our understanding to establish new therapeutic strategies. Further researches are deserved to resolve the problems associated with the contradictory research results with conflicting roles of different members of miRNA-17-92 due to use of different models, study designs and disease stages.

## 6. The Oncogenic Relevance of miRNA-17-92 

The miRNA-17-92 cluster, also referred to as Onco-miRNA-1, was first identified as an oncogenic miRNA group due to instances of dysregulation that ultimately progressed into tumorigenesis and malignancy [164, 165]. Aberrant expression of miRNA-17-92 has been found in diverse types of tumors [32, 136], which assures its relevance to its oncogenic property in a wide range of cancers, including malignant lymphoma cell lines [34], lung cancer [166, 167], breast cancer [168], colon cancer [134], colorectal cancer [169], prostate, pancreas, liver, gastric and thyroid [16, 164].

By targeting critical genes in cell cycle progression, apoptosis and angiogenesis in multiple tumors, miRNA-17-92 cluster plays a key role in cancer prognosis (Fig. 3). The miRNA-17-92 represses the tumor suppressor gene, SOCS-1, an endogenous inhibitor of the Janus kinase/signal transducer and activator of transcription (JAK-STAT) pathway [170, 171, 172]. Activation of JAK-STAT signaling promotes cancer cell proliferation by regulating cyclin D1 and inhibits cell apoptosis by regulating, myeloid cell leukemia 1 (Mcl-1) and Bcl2 [170].

The oncogenic role of miRNA-17-92 cluster is attributed in cancer cells by directly targeting PTEN and in consequence activating PI3K/AKT/GSK/mTOR pathway, which promotes cell proliferation and angiogenesis and blocks apoptosis [157, 173, 174]. In esophageal squamous cell, overexpression of miRNA-18a promotes the expression cyclin D1 by targeting PTEN-PI3K-AKT-mTOR signaling [175]. miRNA-19 induces cell proliferation and oncogenic growth by directly targeting PTEN, thus activating the AKT–mTOR signaling and promoting c-myc-induced lymphomagenesis by repressing apoptosis [30]. By regulating PTEN/AKT pathway, miRNA-19a/b promotes multidrug resistance (MDR) in gastric cancer cells by accelerating the efflux of chemotherapeutic drugs and inhibiting drug-induced apoptosis [176, 177]. Different members of the miRNA-17-92 family also suppress IL-12 expression by targeting PTEN to modulate the PI3K-AKT-GSK3 pathway in the immune system [178].

The proto-oncogene Myc stimulates angiogenesis and tumor growth by amplifying miRNA-17-92, which attenuates TGFβ/Smad2/4 signaling pathway and represses antiangiogenic factors TSP-1 and CTGF [134, 135, 179]. Specifically, miRNA-20 disrupts TGFβ-mediated p21/Myc regulation in many malignancies [180]. Another transcription factor, *E2F1* is also widely involved in tumorigenesis under the regulation of *Myc* [181]. Clinical studies revealed that enhanced levels of *E2F*/*Myc* and different members of miRNA-17-92 cluster are positively correlated with invasiveness and proliferation of pituitary neuroendocrine tumors and pediatric brain tumors, which could be potential biomarkers of tumors [182, 183]. A truncated version of the cluster, miRNA-17-19b protects Myc-driven B cell lymphomagenesis by fine-tuning Myc expression. The miRNA-17/20 targets the checkpoint kinase 2 (Chek2 kinase) with subsequent reduction of HuR phosphorylation, which increases recruitment of human antigen R (HuR) (RNA binding protein) to Myc mRNA, ultimately inhibits Myc translation and sustains unlimited tumor growth [184]. Using a mathematical model for the Myc/E2F1/miRNA-17-92 network, Sengupta *et al*. [185] predicted that miRNA-17-92 plays a decisive role toward cellular proliferation or apoptosis or even quiescence by controlling the *Myc*/*E2F* in different cancers. In lung cancer, miRNA-17-92 facilitates cell proliferation and survival by suppressing the expression of HIF-1α and c-Myc transcriptional activity [186].

The multifaceted roles of the miRNA-17-92 cluster have been identified in colorectal cancer biology. Several studies reported that elevated expression of miRNA-17-92 cluster is associated with invasion and metastasis of colorectal cancer cells [187, 188]. The uncontrolled expression of miRNA-17-92 in colorectal cancer activates oncogenic signaling pathways, particularly the WNT/β-catenin pathway [189, 190]. The miRNA-92a promotes WNT/β-catenin signaling activity by directly targeting KLF4, GSK3β, and Dickkopf-3 (DKK3), which subsequently enhances chemoresistance of colorectal cancer cells as well as stem cell-like phenotypes to induce colorectal cancer cell progression [190]. The miRNA-92 also promotes lymph node metastasis of colorectal cancer patients by suppressing PTEN expression and consequently activation PI3K/AKT pathway [191]. Similarly, elevated expression of miRNA-17 is related to liver metastasis and shorter survival of colorectal cancer patients [188, 192].

On the contrary, tumor suppressive role of miRNA-17-92 has also been identified in certain cancer types [32, 193]. Reduced expression of different members of miRNA-17-92 cluster is detected in ovarian cancers, melanomas, and breast cancers [194]. O’Donnell *et al*. [135] reveals that miRNA-17 and miRNA-20 function as a tumor suppressor in the human B cell line by suppressing Myc-induced *E2F1* expression, thereby inhibits Myc-mediated cellular proliferation. Interestingly, an overexpression of another member of the cluster, miRNA-92, leads to aberrant increase of *Myc*, which stimulates excessive cell proliferation as well as p53-dependent apoptosis in B-cell lymphomas [195]. In cervical cancer, miRNA-17- 92 directly suppresses Cdt2 (cell cycle S phase licensing factor, CDC-10 dependent transcript-2) and blocks the cancerous cells in S phase and induces apoptosis, without affecting non-cancerous cells [196]. Using a semi-high-throughput *in vivo* screening platform using hyperactive piggyBac (hyPB) transposons system to functionally screen miRNA-17-92 cluster members *in vivo* mouse livers, Tipanee *et al*. [197] demonstrates that miRNA-20a acts as a tumor suppressor gene in hepatocellular carcinoma models.

Tumor suppressive role of miRNA-18 is exhibited in colorectal cancer cells [198]. Lower expression of miRNA-18 is detected in colorectal tissues of both early and advanced colorectal cancer patients. Directly targeting CDC42 (cell division cycle 42, a mediator of the PI3K pathway) and cell-cycle progression gene, *CCND1*, miRNA-18a reduces colorectal cancer cell growth. The miRNA-18a also represses the proliferation of bladder cancer cells by targeting Dicer 1 expression, a gene required for miRNA biogenesis, which has an active role in tumorigenesis and cancer progression [199]. Oncogenic roles of miRNA-18a have also been reported in different cancers, including lung cancer, cervical cancer, prostate cancer and gastric cancer [200, 201, 202, 203]. The miRNA-18a promotes lung cancer cell proliferation and migration by targeting interferon regulatory factor 2 (IRF2), an IRF family member, that plays a crucial role in adaptive immunity in tumorigenesis [202]. In cervical cancer, miRNA-18a induces PD-L1 (programmed death–ligand 1) expression by targeting PTEN, with-no-lysine kinase 2 (WNK2) (ERK1/2 inhibitor), and SRY-related HMG-box transcription factors (SOX) 6 (WNT/β-catenin inhibitor) with activation of PI3K/AKT, MEK/ERK, and WNT/β-catenin pathways [181]. In prostate cancer cells, miRNA-18a enhances AKT phosphorylation by targeting STK4 (serine/threonine-protein kinase 4), which promotes cell survival and tumorigenesis [201]. By targeting sterol regulatory element binding transcription protein 1 (SREBP1), miRNA-18a decreases the lung metastasis of breast cancer, which form a co-repressor complex with SNAIL and histone deacetylase 1/2 (HDAC1/2) to modulate EMT (epithelial-mesenchymal transition) [204].

The beneficial prognostic role of miRNA-19b for patients with hepatocellular carcinoma has been revealed by Hung *et al*. [205]. In hepatocellular tumors, miRNA-19b is significantly overexpressed compared with adjacent non-tumor liver tissues [205], which is correlated with better disease-free survival of the patients with hepatocellular carcinoma. The study demonstrates that miRNA-19b regulates several genes involved in the metastasis process, including HIF-1α and mitogen-activated protein kinase 14 (MAPK14) in tumors of patients with human hepatocellular carcinoma.

In highly migrated oral squamous carcinoma cells, miRNA-17, miRNA-19, miRNA-20, and miRNA-92 are significantly down-regulated and overexpression of the miRNA-17/20 suppresses the migratory ability of these cells by targeting integrin (ITG) β8 [206]. The miRNA-17 and miRNA-20a expression levels are also negatively correlated with an advanced tumor stage and lymph node metastasis status in patients with oral cancer [206].

Anti-oncogenic and stimulating drug-sensitivity functions of miRNA-17-92 cluster have also been reported in prostate cancer and breast cancer [207, 208, 209]. In prostate tumor tissue of cancer patients and in aggressive prostate cancer cells, the expression of miRNA-17-92 is significantly downregulated. Restoration of miRNA-17-92 suppresses the expression of different cell cycle regulatory proteins (cyclin D1, slingshot protein phosphatase 1 (SSH1)), actin cytoskeleton reorganization (LIM domain (Lin-11, Isl-1, and Mec-3) kinase 1 (LIMK1)), RhoGTPase pathway (FYVE, RhoGEF And PH domain containing 4 (FGD4)), which decreases cell proliferation, reduces activation of AKT and microtubule-associated proteins (MAP) kinases, delays tumorigenicity and tumor growth in prostrate tumor-bearing mice [208]. Similarly, the abundance of miRNA-17/20 expression is reduced in highly invasive breast cancer cell lines and node-positive breast cancer specimens. Conditioned medium from miRNA-17/20 overexpressing breast cancer cells inhibits invasion and metastasis of breast cancer by inhibiting the secretion of growth promoting cytokines [209]. Moreover, differential expression of miRNA-17-92 in diverse types of breast cancer is reported by Hossain *et al*. [207]. The expression of miRNA-17-92 is elevated in triple negative breast cancer (TNBC) with poor outcome. However, its expression is reduced in estrogen receptor (ER)-positive breast cancer (ERPBC). Ectopic expression of miRNA-17-92 in ER-positive breast cancer cells represses cell growth, migration and invasion, as well as enhances chemosensitivity. The study indicated that expression of the adenosine A1 receptor (ADORA1) is repressed by miRNA-17-92 in ERPBC. Intriguingly, by repressing cyclin D1 and its binding protein amplified in breast cancer 1 (AIB1) expression, miRNA-17 and miRNA-20 suppress breast cancer cell proliferation and breast tumorigenesis [193, 210].

## 7. Biomarker/Therapeutic Target in Cancer

Numerous studies consistently suggest that circulating miRNAs may be the promising diagnostic as well as prognosis biomarkers of various cancers [211, 212]. Several members of miRNA-17-92 are differentially expressed in the plasma of patients with cancer. The miRNA-17-3p and miRNA-92 were significantly upregulated in patients with colorectal cancer than healthy controls, suggesting potential diagnostic markers for colorectal cancer [213, 214]. Multiple studies report that serum levels of miRNA-17, miRNA-18a, and miRNA-20 are elevated in gastric cancer patients in comparison to healthy controls [215, 216, 217, 218, 219]. Deregulation of circulating miRNA-17 in serum samples from breast cancer patients may be relevant for different types of breast cancer development, progression, and metastasis [220]. Prognostic significance of serum miRNA-17-5p was also identified in the patients with lung cancer by Chen *et al*. [221]. In serum of patients with lung cancer, the expression of miRNA-17-5p was significantly increased in compared with healthy individuals and lower serum level of miRNA-17-5pexpression was correlated to the survival of patients. Although numerous studies proposed the circulating miRNA-17-92 as potential diagnostic and prognostic biomarkers of malignancies, no miRNAs either in blood or solid tumors have been approved for clinical purposes due to the inconsistency of expression patterns of these miRNAs in different studies.

## 8. Conclusion 

As discussed, the members of miRNA-17-92 cluster serve as the key regulators of many cellular processes, overseeing the maintenance of normal development, regulation of cell cycle machinery, proliferation, the response to different stimuli, pathological conditions and aging. Several pioneering studies have revealed this cluster’s critical role in multiple diseases and malignancies, its precise roles demand further exploration. Several studies explore the differentially dysregulation of multiple members of miRNA-17-92 with molecular mechanisms in heart and cancer, which deserve further investigation. Unbalanced expression of different members of miRNA-17-92 in multiple cellular contexts pose negative implications, spurring development of numerous pathological and physiological functions spanning multiple organ systems. Numerous studies have established both oncogenic and tumor suppressor roles of miRNA-17-92 cluster. As an oncogene, miRNA-17-92 regulates coordinated multiple cellular processes to achieve malignant transformation, causes reduced cancer cell death and apoptosis, rapid cell proliferation, and increased angiogenesis. However, overexpression of several members of miRNA-17-92 suppressed cell proliferation, adhesion, and migration in certain cancer. As cardiovascular diseases and cancer continue to plague the global population, advancing our understanding of the diverse fundamental roles of the miRNA-17-92 cluster is a crucial step in further development of diagnostic, prognostic and therapeutic tools. Despite great progress in understanding the several key roles of miRNA-17-92, to ensure its clinical application for disease prevention, clinical diagnosis, prognosis, and targeted therapy, a thorough exploration of miRNA-17-92’s multifaceted roles and underlying mechanisms of actions is imperative.
